# Monoclonal Antibody Therapy and Renal Transplantation: Focus on Adverse Effects

**DOI:** 10.3390/toxins6030869

**Published:** 2014-02-28

**Authors:** Gianluigi Zaza, Paola Tomei, Simona Granata, Luigino Boschiero, Antonio Lupo

**Affiliations:** 1 Renal Unit, Department of Medicine, University-Hospital of Verona, Piazzale A. Stefani 1, Verona 37126, Italy; E-Mails: paola.tomei@libero.it (P.T.); simona.granata@univr.it (S.G.); antonio.lupo@univr.it (A.L.); 2 First Surgical Clinic, Kidney Transplantation Center, University-Hospital of Verona, Piazzale A. Stefani 1, Verona 37126, Italy; E-Mail: luigino.boschiero@ospedaleuniverona.it

**Keywords:** renal transplantation, adverse effects, toxicity, Basiliximab, Rituximab, Eculizumab, malignancy, infection, toxicity

## Abstract

A series of monoclonal antibodies (mAbs) are commonly utilized in renal transplantation as induction therapy (a period of intense immunosuppression immediately before and following the implant of the allograft), to treat steroid-resistant acute rejections, to decrease the incidence and mitigate effects of delayed graft function, and to allow immunosuppressive minimization. Additionally, in the last few years, their use has been proposed for the treatment of chronic antibody-mediated rejection, a major cause of late renal allograft loss. Although the exact mechanism of immunosuppression and allograft tolerance with any of the currently used induction agents is not completely defined, the majority of these medications are targeted against specific CD proteins on the T or B cells surface (e.g., CD3, CD25, CD52). Moreover, some of them have different mechanisms of action. In particular, eculizumab, interrupting the complement pathway, is a new promising treatment tool for acute graft complications and for post-transplant hemolytic uremic syndrome. While it is clear their utility in renal transplantation, it is also unquestionable that by using these highly potent immunosuppressive agents, the body loses much of its innate ability to mount an adequate immune response, thereby increasing the risk of severe adverse effects (e.g., infections, malignancies, haematological complications). Therefore, it is extremely important for clinicians involved in renal transplantation to know the potential side effects of monoclonal antibodies in order to plan a correct therapeutic strategy minimizing/avoiding the onset and development of severe clinical complications.

## 1. Role and Biological Functions of Monoclonal Antibody Therapy in Renal Transplantation

Renal transplantation has been a major breakthrough in the treatment of end-stage renal disease (ESRD) by improving quality of life and reducing the mortality risk for most patients, when compared with maintenance dialysis [1]. However, renal allograft recipients still have a high mortality rate compared with the general population. 

In addition, in the last years, there has been a significant improvement in short-time graft survival by ameliorating organ preservation, surgical techniques, postoperative care, and, in particular, by introducing more effective immunosuppressive drugs [2]. Recent literature evidence shows that one-year renal allograft survival has increased from 50% to nearly 90% when cadaveric donors and to 95% when living donors are used [3,4,5]. This success has been also achieved by providing a high degree of immunosuppression at the time of transplantation utilizing several induction therapy protocols. 

The use of antibody induction therapy has increased dramatically over the last 20 years [6]. Prior to 1993, fewer than 30% of renal transplantations were performed with induction therapy and, currently, it is utilized in over 80% of renal transplantations [7].

This therapeutic strategy, initiated intraoperatively or immediately postoperatively, has the main objective to reduce the incidence of early acute rejections [8], historically known to predict early graft loss [9] in particular in renal transplant recipients at high risk for poor short-term outcomes, such as patients with preformed antibodies, history of previous organ transplants, multiple human leukocyte antigen mismatches, or transplanted organs with a prolonged cold-ischemic time or from expanded-criteria donors [10].

Induction therapy in renal transplant recipients has also the aim to decrease the incidence of delayed graft function (DGF) [11], mitigate the impact of DGF by reducing the incidence of acute rejection, and allow immunosuppressive minimization preventing calcineurin inhibitor (CNI)-induced nephrotoxicity immediately after transplant surgery [12]. By using induction therapy, initiation of CNI therapy can often be delayed until the graft regains some degree of function [12,13,14].

**Figure 1:**
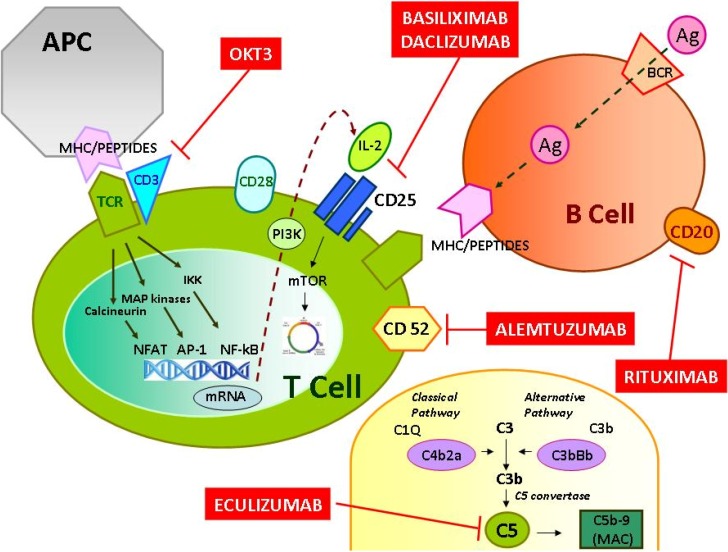
Sites of action of available monoclonal antibodies in renal transplantation. Basiliximab and daclizumab bind with high affinity to the interleukin-2 receptor (CD25) and prevent the formation of the IL-2 binding site interrupting the cascade of cellular events leading to cell activation, proliferation and cytokine release. Alemtuzumab is directed against the cell surface glycoprotein CD52, a peptide present on the surface of mature lymphocytes, determining an antibody-dependent lysis of lymphocytes. Rituximab induces cytotoxicity by binding the CD20 antigen located on the surface of B-cell. Eculizumab is directed against the complement protein C5, thereby inhibiting conversion of C5 to C5b and preventing formation of the membrane attack complex (C5-9). OKT3 is an immunoglobulin that targets the CD3 protein on the surface of circulating human T cells, which is part of the T-cell receptor complex. Thus, OKT3 blocks both the generation and function of cytotoxic T cells clearing them from the circulation.

The majority of these medications, targeted against specific CD proteins on the T or B cells surface (e.g., CD3, CD25, CD52) (Figure 1), have a primary role in the control of cellular and humoral immune system activation that provides a significant barrier to solid organ transplantation through a direct effects of cytotoxic/effector cells or indirectly by an antibody-mediated recognition of non-self proteins and carbohydrates expressed on transplanted organs [15,16]. 

Then, in the last few years, the use of monoclonal antibodies has been proposed for the treatment of chronic antibody-mediated rejection (CAMR), the major cause of late renal allograft loss [17]. 

## 2. Adverse Effects/Toxicities of the Monoclonal Antibody Therapy in Renal Transplantation

At the moment, there is no universal consensus on the optimal monoclonal antibody for induction therapy and/or for the treatment of acute rejection. However, most of the time the medical decision needs to take into account the balance between benefits (e.g., reduction of acute rejection rate), costs, and risks to develop morbidities.

**Figure 2:**
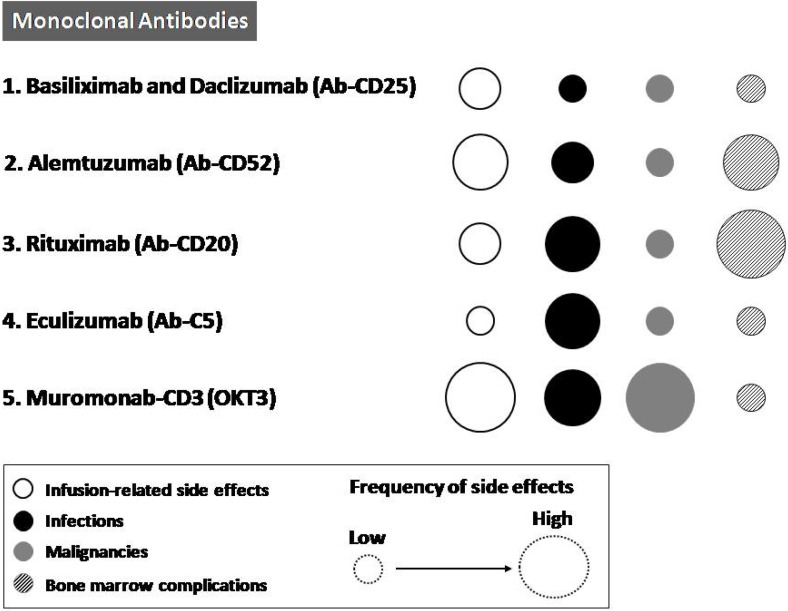
Schematic representation of the major side effects frequency of basiliximab and daclizumab (Ab-CD25), alemtuzumab (Ab-CD52), rituximab (Ab-CD20), eculizumab (Ab-C5), and muromonab-CD3 (OKT3). White dots symbolize infusion-related side effects, black dots infections, gray dots malignancies and white and black striped dots bone marrow complications. As indicated in the box below, dot size is representative of the side effects frequency.

In fact, it is unquestionable that by using these highly potent immunosuppressive agents, the body loses much of its innate ability to mount an adequate immune response, thereby increasing the risk of infectious complications and malignancies [12,14,18,19] (Figure 2). 

In this review, we analyzed the available literature evidence, reporting the adverse effects associated with the treatment of the most commonly used monoclonal antibodies in renal transplantation: anti-CD25 (Basiliximab and Daclizumab), anti CD-52 (Alemtuzumab), anti-CD20 (Rituximab), anti-C5 (Eculizumab), and anti-CD3 (OKT3).

## 3. Non-Depleting Antibodies

### 3.1. Anti-CD25 Mechanism of Action

Basiliximab (Simulect, Novartis; Novartis International AG, Basel, Switzerland) and daclizumab (Zenapax; Roche Pharmaceuticals, Nutley, NJ, USA) are humanized monoclonal antibodies anti-IL-2-R (or anti-CD25) frequently used in renal transplantation as induction therapy agents [20]. Basiliximab is a chimeric monoclonal antibody, while daclizumab (90% human, 10% murine) is a humanized antibody built by total gene synthesis using oligonucleotides. 

Both these agents bind with high affinity to the 55 kD alpha-chain of the interleukin-2 receptor (IL-2-R or CD25) and prevent the formation of the IL-2 binding site [21]. Heterotrimerization and participation of the IL-2-R-alpha-chain in the IL-2-R complex confers high-affinity binding properties to IL-2 with subsequent rapid clonal expansion of activated T-lymphocytes [22,23]. Specific inhibition of T cell activation by IL-2-R α-chain antibodies could interrupt the cascade of cell activation, proliferation and cytokine release which lead to tissue inflammation and acute rejection [24].

Based on their peculiar biological/pharmacological characteristics, they have been largely studied and used in renal transplantation causing a significant reduction in the acute rejection rate and allowing a CNI and steroids minimization in the early postoperative period after renal transplantation [25,26,27].

In particular, basiliximab has been approved by the FDA only for transplant induction therapy in 1998 and is currently used in renal transplant patients with low immunological risk [28,29,30]. In fact, as reported by KDIGO (Kidney Disease: Improving Global Outcomes), although rabbit anti-thymocyte globulin (rATG) is superior to anti-CD25 in high-risk transplant recipients, the potential risks of infection and malignancy outweigh these benefits in standard-risk recipients [30]. 

Additionally, as extensively demonstrated, these drugs have been successfully used in high risk patients with other medical comorbidities that preclude usage of lymphocyte-depleting antibody safely. In fact several clinical reports have showed that basiliximab induction is safe and adequate for renal transplant, also including the high risk transplants, such as deceased donor kidney transplants in highly sensitized African Americans [31], particularly in simultaneous kidney pancreas transplant [32], and splitting single pediatric donor kidney transplant [33], as long as the conventional triple regimen consisting of calcineurin inhibitors, mycophenolic acid, and steroids.

Although having similar efficacy, the dosing regimen for basiliximab consists of two 20-mg doses, one administered two hours before transplantation and the second on day four posttransplant, whereas daclizumab is usually administered as five doses over eight weeks [34]. This simple and convenient regimen provides suppression of the IL-2 receptor for 30 to 45 days, when the risk of acute rejection is the greatest [35]. 

During time, this difference in administration regimen has led to a more frequent use of basiliximab than daclizumab. For this reason, daclizumab has been withdrawn from the market.

### 3.2. Anti-CD25 Side Effects

Although, anti-CD25 has the best safety profile compared to other available induction antibodies [34,36,37], they can still enhance the risk of developing important adverse effects/toxicities.

*Hypersensitivity reactions:* These rare complications (occurring in less than 1% of treated patients), may determine several clinical effects: hypotension, tachycardia, cardiac failure, bronchospasms, pulmonary edema, and respiratory failure [34,35,36]. The exact biological mechanism involved is not completely defined. 

To avoid hypersensitivity reactions, sometimes life-threatening, extreme caution should be used in all patients previously treated with these drugs.

*Infectious complications:* Perioperative induction therapy with anti-CD25 may induce higher risk of bacterial, viral, and fungal infections as compared with placebo [38]. However, the cumulative infection risk is significantly lower in anti-CD25-treated patients compared to those undergoing a more aggressive anti-thymocyte globulin (ATG) induction therapy [38,39,40,41]. 

In particular, Brennan *et al.* have reported that the incidence of overall infection was higher in the ATG group than in the basiliximab group (85.8% *vs.* 75.2%, *p* = 0.03). This difference appeared to be attributable to a greater frequency of urinary tract infections (39.0%, *vs.* 27.0%; *p* = 0.04) and non-cytomegalovirus (CMV) viral infections (21.3% *vs.* 11.7%, *p* = 0.04) in the ATG group. On the contrary, the incidence of CMV infection resulted lower in the ATG group as compared with anti-CD25 (7.8% *vs.* 17.5%, *p* = 0.02) [38]. 

*Malignancies*: Malignancies or post-transplant lymphoproliferative disorders are rare in anti-CD25 treated renal transplant patients [42] and less frequent as compared with ATG-treated patients [38,43].

*Myelosuppression*: It has been reported that, basiliximab-treated renal transplant patients may experience leukopenia (approximately 10%–15%) and thrombocytopenia (5%). Anyway, the incidence of these adverse effects resulted most of the time lower as compared with that evaluated in ATG-treated patients [38,44].

### 3.3. Rabbit Antithymocyte Globulin (rATG) *versus* BASILIXIMAB: Just a Few Words

It is largely reported that rabbit antithymocyte globulin (rATG, Thymoglobulin™, Genzyme Canada Inc., Mississauga, ON, Canada), together with basiliximab, are the most widely used antibodies in renal transplantation. 

This agent is a polyclonal gamma immunoglobulin derived from the immunization of rabbits with human thymocytes and indicated for the prevention and treatment of acute renal transplant rejection [45]. 

Induction with rATG, together with maintenance immunosuppression, has been shown to be more effective than maintenance immunosuppression alone in preventing episodes of acute rejection in adult renal transplant recipients [46,47]. 

Additionally, results of a large clinical trial, using moderate to high-risk deceased donor recipients, demonstrated an improved composite endpoint of the incidence of rejection, graft loss, and patient death that favored rATG *versus* basiliximab [38,48]. At a mean of 10 months of follow-up, the estimate of combined endpoint was 19.1% in the rATG arm and 31.6% in the basiliximab arm (*p* = 0.01), with acute rejection being the driving factor (14.2% rATG *vs.* 25% basiliximab, *p* = 0.013). Five-year follow-up demonstrates that the incidence of acute rejection requiring antibody rescue remained lower in the rATG group (3% *vs.* 12%, *p* = 0.05). 

However, adverse events, such as fever, chills, and gastrointestinal distress, are more frequent with rATG than with other induction agents [38,39]. Serious reactions, such as cytokine release syndrome with hemodynamic instability, can also occur and are most commonly associated with the first dose and rapid infusion rates [45]. Finally, rATG may induce severe CMV infections [39,46,49,50,51,52].

## 4. Depleting Antibodies

### 4.1. Mechanism of Action of Alemtuzumab (Anti-CD52 Antibody)

Alemtuzumab (MabCampath or Campath; Genzyme, Cambridge, MA, USA) is a novel recombinant DNA-derived humanized rat monoclonal antibody directed against the 21–28 kDa cell surface glycoprotein CD52 [53]. Alemtuzumab was never approved for use in transplantation and no Phase II/III trials have been performed. Furthermore, this drug has been withdrawn from general marketing, not out of safety or efficacy concerns, but rather to reposition the drug for use in multiple sclerosis.

Human CD52 is a peptide of 12 amino acids, anchored to glycosylphosphatidylinositol, present on the surface of mature lymphocytes (including T and B lymphocytes, macrophages, monocytes, and natural killer cells), but not on on lymphoid progenitors [54]. It is also found on monocytes and dendritic cells. 

Although the exact underlying mechanism remains unclear, alemtuzumab has been shown to produce significant leukopenia by means of antibody-dependent lysis of lymphocytes, leading to depletion of T and B cells in the peripheral circulation for several months after administration [54,55]. 

Alemtuzumab was initially used as an anti-cancer therapy for the treatment of B-cell chronic lymphocytic leukaemia, but, in the last few years, its use as an induction therapy following solid organ transplantation (off-label) is increasing world-wide [56]. 

Accordingly, a number of clinical trials have been undertaken to evaluate its efficacy and safety in kidney transplantation with contrasting results that make difficult to reach a consensus on its application [57,58,59,60,61].

### 4.2. Alemtuzumab Side Effects

Results from clinical reports and trials focusing on renal transplantation have demonstrated that, although effective, alemtuzumab may induce severe adverse effects.

*Infusion related side effects*: Because of the xenogenic nature of the antibody, adverse reactions can occur during or shortly after alemtuzumab infusion (generally first week of treatment). They include: pyrexia, chills/rigors, nausea, hypotension, urticaria, dyspnea, rash, emesis, and bronchospasm [62]. 

In some cases, serious, including fatal, infusion reactions have also been identified in post-marketing reports: syncope, pulmonary infiltrates, acute respiratory distress syndrome, respiratory arrest, cardiac arrhythmias, myocardial infarction, acute cardiac insufficiency, cardiac arrest, angioedema, and anaphylactic shock [63]. 

Most of the time, alemtuzumab-associated cytokine release syndrome could be prevented by co-administration of steroids and/or antihistamines.

*Malignancy:* Although present, clinical studies do not report a higher incidence of neoplasia in alemtuzumab-treated patients in particular when compared to other induction regimens.

As described by Puttarajappa C *et al.* the incidence of malignancies observed during a median follow up of four years was 2.8%. However, alemtuzumab induction was not associated with a significant increased post-renal transplant cancer risk when compared to no induction therapy and it was associated with lower cancer incidence when compared to thymoglobulin. Authors of this paper underlined that the relatively low observed predisposition to induce cancer could be mainly due to CNI minimization [64]**.**

Additionally, the group of Hanaway described a very low incidence of B-cell lymphoma (<1%) and skin cancer (4%) in patients treated with alemtuzumab during a follow-up period of three years [65]. 

Moreover an analysis of the data collected by the Organ Procurement and Transplantation Network/United Network for Organ Sharing (OPTN/UNOS) demonstrated no association between alemtuzumab treatment and risk for post-transplant lymphoproliferative disorder (PTLD) [66].

*Infection*: It is indisputable that almetuzumab, as other immunosuppressive induction agents, may increase the incidence of infections in renal transplant recipients. 

A recent study has reported a frequency of CMV infection of 13%, BK virus infection of 11%, Herpes simplex of 3%, and Herpes Zoster of 5% in anti-CD52 treated patients [65]. 

However, unlike bone marrow and liver transplant recipients, and hemo-oncological patients [67], no strong evidence supports the association between infection risk and almetuzumab treatment in renal transplant recipients. In fact, only one randomized controlled trial clearly reported a significant difference in the risk of CMV infection in alemtuzumab treated patients as compared with controls no receiving induction immunosuppression (28% *vs.* 12%, *p* = 0.03) [58].

The relatively low incidence of CMV infections in renal transplant patients treated with anti-CD52 could be mainly due to the use of a well standardized CMV viral prophylaxis (using ganciclovir or valganciclovir) in high risk patients (recipients of CMV-positive donors) and in an effective world-wide adopted pre-emptive treatment strategy. 

*Bone marrow disease:* In a phase 2 clinical trial the treatment of relapsing-remitting multiple sclerosis with alemetuzumab, six of 216 patients (2.8%) developed immune thrombocytopenia (ITP), strikingly higher than the incidence rate of ITP reported for the general adult population. They describe an unique form of ITP associated with alemtuzumab treatment and characterized by delayed presentation after drug exposure, responsiveness to conventional ITP therapies, and prolonged remission. The pathogenesis of alemtuzumab-associated ITP is incompletely understood. The authors suggested that alemtuzumab-associated ITP may arise as a consequence of defects in central tolerance checkpoints during lymphocyte reconstitution. With immune recovery, resolution of these defects may contribute to the favorable natural history of the disorder [68]. 

In addition, Reda G *et al.* reported an increase incidence of ITP in patients affected by chronic lymphocytic leukemia treated with low-dose alemtuzumab. ITP was diagnosed in 12 (18.7%) patients: nine (14%) patients developed ITP during or after treatment with alemtuzumab (median observation time 30 months, range 9–23), whereas, in the remaining three patients, it preceded the treatment [69]. This data, associated with the report of Cuker *et al.*, suggest an important role of alemtuzumab in the pathogenesis of ITP, which could be related to its ability to induce dysregulation of T-lymphocyte activity. 

Hanaway *et al.* describe among low-risk patients a mean total lymphocyte count significantly lower in patients treated with alemtuzumab than basiliximab. Among high-risk patients, the use of lymphocyte-depleting antibodies (alemtuzumab and ATG) in both treatment groups resulted in a low mean total lymphocyte count within the first week after transplantation to less than 10% of the baseline value [65].

### 4.3. Rituximab (Anti-CD20/ Anti-B-Cell) Mechanism of Action

Rituximab is a chimeric anti-CD20 monoclonal antibody licensed for use in non-Hodgkin’s lymphoma (NHL), chronic lymphocytic leukaemia, and rheumatoid arthritis [70]. At the moment, rituximab is not approved for use in renal transplantation and it is used on an off label basis. No phase II or phase III studies have been performed yet and the published data are still controversial. The CD20 antigen is a transmembrane nonglycosylated phosphoprotein, expressed on both immature and mature B cells, involved in calcium conductance and regulation of cell proliferation/differentiation [71]. 

Once rituximab has bound to the CD20 antigen, it affects B cells by inducing [72]: (1) activation of the complement cascade, leading to complement-mediated cytotoxicity; (2) macrophage recognition, inducing phagocytosis and antibody-dependent cell-mediated cytotoxicity (ADCC); and (3) natural killer cell interaction, also leading to ADCC. It does not have a direct effect on plasma cells (which do not express the CD20 antigen).

Additionally, rituximab causes a decrement of the number of B cells present in the peripheral blood circulation within one to three days of administration, and complete B cell depletion in most of the patients within one to six weeks [73].

Although, never licensed for use in renal transplantation, this agent has been frequently used in several induction protocols for HLA antibody incompatible transplantation and for the treatment of acute renal allograft rejections, CAMR and PTLD [74,75,76,77,78].

### 4.4. Rituximab Side Effects

Even if generally considered a safe agent, rituximab may induce several dose- and/or time-related adverse effects. Early recognition of them could help clinicians to plan a prophylaxis strategy and/or start a treatment in order to avoid the development of severe clinical complications.

The most frequent adverse effects associated with rituximab treatment are the following:

*Infectious complications*: Although debated, several studies have suggested that rituximab may slightly increase the risk of infection (including Pneumocystis pneumonia, Hepatitis B, and CMV disease and fungal infection) [79,80].

Additionally, during a three-year follow-up of ABO-incompatible renal transplant recipients treated with antigen-specific immunoadsorption and rituximab reported an incidence rate of sepsis (6.7%), urinary tract infection (13%), and surgical wound infection (13%) [81]. In the same population, the incidence of CMV infection was 6.7% and none of the patients developed EBV, BKV associated nephropathy, invasive fungal infections, and Clostridium difficile colitis. 

Additionally, however, as with other immunomodulators, the current label for rituximab contains a black box warning about the risk of re-activation of hepatitis B virus (HBV) infection, although the exact impact of rituximab on the incidence of HBV re-activation in patients with current or past HBV infection remains largely unknown [82]. 

At present, it is well known that the above mentioned risk of HBV re-activation is quite high among HBV carriers, particularly if rituximab is given alone or in combination with steroids [83,84]. The issue is much more complex in patients with serological markers of past HBV infection [HBsAg negative with positive antibodies against the HBV core antigen (anti-HBc positive)] undergoing either conventional chemotherapy or rituximab-based therapy [85].

For instance, the EASL clinical practice guidelines for the management of chronic HBV infection in HBsAg-negative patients with positive anti-HBc antibodies who receive chemotherapy and/or immunosuppression suggest HBV-DNA determination in the serum and if undetectable, strict follow-up by means of alanine aminotransferase (ALT) and HBV-DNA testing [86]. 

Treatment with potent antivirals having a high barrier to resistance (e.g., entecavir or tenofovir) is recommended upon confirmation of HBV re-activation before ALT elevation [86].

However, there is no clear evidence for when and how often this “strict” follow-up should be performed as surrogate or prognostic markers related to the development of HBV re-activation in these subjects are obscure and the cost effectiveness particularly of HBV-DNA serial testing is unknown.

However, further studies, designed explicitly to assess the impact of rituximab on infection rates, are required.

*Non-infectious pulmonary toxicity*: A systematic literature review reported several cases of rituximab-associated interstitial lung disease (RTX-ILD) in non-renal transplant patients [87,88]. A total of 121 cases of potential RTX-ILD were identified from 21 clinical studies/trials. In 30 (24.7%) cases it was given as monotherapy. The mean time of onset, from the last infusion until symptoms development or relevant abnormal radiological change was 30 days.

Radiological findings were characterized by diffuse bilateral lung infiltrates apparent on chest radiographs and/or thoracic computed tomography (CT).

In all cases, rituximab was discontinued and corticosteroids were administered immediately. In patients who improved in association with corticosteroids treatment, symptomatic recovery was achieved within several days, with radiological resolution lagging behind (weeks to months). Lung infiltrates on CT disappeared within a maximum of five months from initial onset. RTX-ILD was fatal in more than 10% of cases. 

Moreover, cases of bronchiolitis obliterans with organizing pneumonia (BOOP) have been reported in patients who received rituximab-based therapy for NHL [89,90].

The mechanisms leading to RTX-induced interstitial lung disease are unknown. 

*Cytokine release syndrome:* Similarly to OKT3, also rituximab may induce cytokine release syndrome [91] particularly in patients with B cell malignancies where the number of CD20+ cells susceptible to this therapy are much greater than in patients with renal failure, who tend to have lower numbers of B cells [92]. The effects of a cytokine release syndrome can be preempted by prophylactic administration of paracetamol, steroid, and antihistaminic drugs.

*Haematological adverse effects:* Neutropenia and thrombocytopenia are the main haematological complications occurring in rituximab-treated patients [93,94]. 

The mechanisms underlying the development of neutropenia are not clear, and it has only rarely been seen in solid organ transplantation.

Rituximab treatment depletes the normal B-lymphocyte population, which recovers within three to nine months. During recovery, the acquisition of a new immune repertoire may determine the transient production of autoantibodies, some of which may be directed against neutrophils or hematopoietic precursors [95]. 

Another hypothesized mechanism includes perturbation of stromal-derived factor 1 levels at the time of B-cell recovery that may interfere with neutrophil egress from the bone marrow and cause neutropenia [96].

Rituximab related late-onset neutropenia has been reported [95] in single patients or small case series of patients receiving rituximab for acute antibody mediated rejection. Ishida *et al.* reported that a significant number of ABO-incompatible and HLA-incompatible transplant patients (approximately 42%) developed late-onset neutropenia (grades III to IV) after the last administration of rituximab [97].

Acute thrombocytopenia is a rare, self-limiting complication following rituximab administration, which is unlikely to lead to bleeding [98]. It may be due to the number of pre-treatment circulating B cells and the onset of cytokine release syndrome [98,99,100].

*Progressive multifocal leukoencephalopathy:* Progressive multifocal leukoencephalopathy (PML) is a rare, serious, and usually fatal demyelinating disease that occurs predominantly in severely immunosuppressed patient populations [101]. Rituximab appears to be responsible of PML, but the causal relationship is unclear. 

The etiologic agent is the JC virus (JCV), a polyomavirus that is widely distributed as a latent infection in the general population but becomes activated, leading to destruction of myelin-producing oligodendrocytes. Patients that develop this complication may present devastating neurologic sequelae. 

However, to our knowledge, no cases of PML in renal transplant patients have been reported so far.

*Rituximab-induced coagulopathy:* Disseminated intravascular coagulation (DIC) appears to be an extremely rare side effect of rituximab. It is a self-limiting event occurring early after the beginning of rituximab treatment. 

To date, this phenomenon has been observed only in patients with WM (Waldenström macroglobulinemia) or HCL (hairy cell leukemia). 

Coagulopathy occurred early after the administration of rituximab and was associated with clinically significant bleeding in half of the cases. The platelet count may drop to approximately 20% and D-dimer values could result frequently elevated. The treatment of this complication is the substitution of either fresh frozen plasma or platelets, or both [102]. 

*Rituximab and cardiovascular complications:* An increased cardiovascular risk has been reported in randomized controlled trials examining the use of rituximab as induction therapy in renal transplantation. In particular, in a recent trial after a three-year follow-up, six of 44 patients died from myocardial infarction. None of 47 patients in the placebo group died. When examined on an intention-to-treat basis (*i.e.*, using death rates from the original cohorts of 68) the difference in mortality was statistically significant (*p* = 0.006) [103].

Therefore, even whether formal guidelines are missed, rituximab should probably be avoided in patients with important cardiovascular comorbidities. Additionally, cardiac tests should be routinely performed in all renal transplant patients prior to rituximab administration.

### 4.5. Muromonab-CD3 (OKT3) Mechanism of Action

Muromonab CD3 (Orthoclone OKT3, Janssen-Cilag, Beerse, Belgium) [104], has been the first monoclonal antibody approved for the treatment of acute rejection and used in several induction protocols for renal transplantation although it has never been formally approved for this indication. 

However, this agent has largely been replaced in transplant medicine by modern induction protocols using ATG characterized by a similar effectiveness and more favourable side-effect profile [105,106].

OKT3 is an Ig2a immunoglobulin produced by hybridoma technique in pathogen-free standard-bred mice that targets the CD3 protein on the surface of circulating human T cells, which is part of the T-cell receptor complex. Thus, OKT3 blocks both the generation and function of cytotoxic T cells clearing them from the circulation. Within 48 hours after OKT3 discontinuation, the normal array of surface molecules including CD3 is found again on all T cells [107]. 

### 4.6. OKT3 Side Effects

Although effective, this drug is characterized by several adverse effects [108,109]: 

*Cytokine release syndrome:* It is an infusion reaction, typically occurring during the first infusion of a new drug, leading to a large range of systemic effects such as flu-like symptoms and, rarely, severe hypotension, bronchospasm, tachycardia, and even death [110]. 

The main biological mechanism leading to this condition is an excessive systemic immune response that involves the release of many different inflammatory mediators, predominantly cytokines. 

Additionally, in most cases this syndrome may be, also, associated with: *nephropathy* as consequence of the enhanced cytokines synthesis and a decrement in the intra-renal prostaglandin synthesis [111]; *pulmonary edema*, due to the concomitant complement-related vascular endothelium damage [112]; *central nervous system (CNS) complications* associated to an activation of T-cells that directly attack specific CNS elements; *activation of coagulation and fibrinolysis* due to complement activation and cytokine release itself leading to graft thrombosis [113,114,115].

Rare life-threatening complications have been also described such as: pulmonary edema, aseptic meningitis, and encephalopathy, opportunistic infections, particularly with cytomegalovirus and post-transplant lymphoproliferative disorders [114,116,117,118,119]. 

*Cancer induction:* OKT3 treatment implies an increased risk of post-transplant malignancies. In particular, because of its specific action against T-cells, it can induce the development of severe lymphoproliferative disorders [108,120,121]. 

*Infectious complications*: The occurrence of CMV and Herpes infections seem to be enhanced in transplanted patients treated with OKT3, especially when this therapy is used in the early phase after transplantation [122].

*OKT3 immunization:* A substantial number (3%–11%) [123] of OKT3-treated patients develop antibodies against the xenogeneic epitope of the agent that are responsible for decreased pharmacological/therapeutic efficacy and increased risk of AMR. In particular, these antibodies can inhibit the binding of OKT3 on CD3 receptor [124]. 

## 5. Monoclonal Antibody Directed against Human Complement Protein

### 5.1. Mechanism of Action of Eculizumab (Anti-C5)

Eculizumab (Soliris^®^, Alexion Pharmaceuticals Inc., Cheshire, CT, USA) is a humanized monoclonal antibody directed against complement protein C5. It binds to the C5 protein with high affinity, thereby inhibiting conversion of C5 to C5b and preventing formation of the membrane attack complex (C5-9) [125]. 

Initially approved for use in paroxysmal nocturnal hemoglobinuria [126,127] and atypical hemolytic-uremic syndrome (aHUS) [128], this agent has been recently used in the treatment of renal allografts recipients with recurrent dense deposit disease and C3 glomerulonephritis [129,130], antibody mediate acute rejections [131,132,133] and prophylaxis of post-transplant recurrence of aHUS [134].

However, no clinical trials involving renal transplant recipients have been performed and the existing data are mainly based on small series and off label use of this agent for the treatment of humoral rejections.

Several authors have reported their experience in the use of eculizumab to prevent acute antibody-mediated rejection after transplantation in a series of HLA-sensitized pre-transplant positive-flow cytometric crossmatch patients [132]. Authors reported that the incidence of this complication at 3 months was significantly lower compared to an historical control group (7.7% *versus* 41.2%), although the presence of C4d in patients with donor-specific antibodies did not differ between the study and control group, thus providing evidence for the downstream activity of eculizumab in blocking the complement pathway. 

Nevertheless, even if this drug is considered a great promising in renal transplantation, at the present, it is rarely used because of the extremely high cost. 

### 5.2. Eculizumab Side Effects

At present, there is still limited but convincing evidence that eculizumab is efficient and well tolerated, but, unfortunately, because of the underutilization of this agent, data are incomplete and not exhaustive.

As reported in the FDA report on the use of eculizumab for the treatment of patients with paroxysmal nocturnal hemoglobinuria [135,136], the more common adverse events associated with eculizumab are: hypertension, diarrhea, headache, anemia, vomiting, nausea, leucopenia, upper respiratory and urinary tract infections, and viral infections (e.g., Herpes simplex virus).

However, as well described, patients treated with eculizumab may develop severe and life-threatening *Neisseria meningitides* infections [137,138]. For this reason is strongly recommended a vaccine therapy against *N. meningitides* before eculizumab administration.

In this context, Struijk *et al.* have described that one transplant patient developed meningococcal sepsis after eculizumab administration despite receiving a polysaccharide meningitis vaccine two weeks beforehand [139]. The patient recovered and showed a humoral response only to *N. meningitidis* serotype C, not to the other constituents of the vaccine (serotypes A, Y, and W135). Moreover, this same group of clinicians has raised concern that the unconjugated polysaccharide vaccine is insufficient as it does not provide protection against serogroup B [140].

Based on the above observations, it is currently recommended that all eculizumab-treated patients should receive a tetravalent (preferably conjugated) vaccine against *N. meningitidis* serotypes A, C, Y, and W135 [104]. Whether it will be sufficient remains to be seen.

## 6. Conclusions

The last decade have witnessed a large employment of monoclonal antibodies in renal transplant clinical practice. They have been utilized as induction therapy, for treatment of steroid-resistant/antibody-mediated acute rejections and chronic antibody mediated-rejections. 

Among them basiliximab has demonstrated a great safety profile and high effectiveness in preventing acute rejection in low risk patients, while the new depleting anti-lymphocyte antibodies rituximab and eculizumab have been useful to prevent/treat transplant-related acute complications particularly in the high risk renal transplant patients.

Additionally, rituximab has demonstrated promising tool to use in the preoperative regimen for the blood group ABO-incompatible transplantation, in desensitization protocols, and for treatment of antibody-mediated rejections. 

Then, eculizumab, interrupting the complement pathway, seems to be highly effective in protecting renal allografts when post-transplant aHUS or AMR occur. Nevertheless, it is unclear how long treatment should continue (a particularly important issue given the high cost of the drug), or whether eculizumab contributes to the development of accommodation in humans. 

However, despite the above described encouraging clinical reports, at the state of art, only few research studies and clinical trials have been undertaken in renal transplantation to really assess the effectiveness of monoclonal antibodies and to measure the clinical impact of these agents on long-term graft and patient survival. 

None of the immunosuppressive regimens using these agents is capable of inducing graft acceptance as one would expect in case of donor-specific tolerance.

Furthermore, no universal consensus exists on the optimal monoclonal antibody dosage and timing. Most of the time the medical decision need to take into account the balance between benefits, costs and risks to develop morbidities.

Therefore, it’s unquestionable that phase II and phase III clinical trials and collaborative research projects should be undertaken to better address the pharmacological potentialities and reduce risks of these agents. In fact, while it is clear their utility, it is also unquestionable that by using these highly potent immunosuppressive agents, the body loses much of its innate ability to mount an adequate immune response, thereby increasing the risk of severe adverse effects (e.g., infections, malignancies, haematological complications). 

A step forward could be also to employ the off-label use of monoclonal antibodies that have originally been developed for other indications.

Finally, we believe that innovative biomolecular or “omics” research strategies could be undertaken to personalize monoclonal antibodies administration in renal transplant recipients [141].
